# A toolset to study functions of Cytosolic non-specific dipeptidase 2 (CNDP2) using *Drosophila* as a model organism

**DOI:** 10.1186/s12863-019-0726-z

**Published:** 2019-03-18

**Authors:** Evgeniya N. Andreyeva, Anna A. Ogienko, Tatiana D. Dubatolova, Anastasiya L. Oshchepkova, Elena N. Kozhevnikova, Anton V. Ivankin, Gera A. Pavlova, Sergei A. Kopyl, Alexey V. Pindyurin

**Affiliations:** 1 Institute of Molecular and Cellular Biology, Siberian Branch of the Russian Academy of Sciences, Novosibirsk, 630090 Russia; 2 Novosibirsk State University, Novosibirsk, 630090 Russia; 3 Institute of Chemical Biology and Fundamental Medicine, Siberian Branch of the Russian Academy of Sciences, Novosibirsk, 630090 Russia

**Keywords:** *Drosophila melanogaster*, CG17337, CNDP2, CN2, CPGL, CRISPR/Cas9, Tumor suppressor gene

## Abstract

**Background:**

Expression of the *CNDP2* gene is frequently up- or down-regulated in different types of human cancers. However, how the product of this gene is involved in cell growth and proliferation is poorly understood. Moreover, our knowledge of the functions of the CNDP2 orthologs in well-established model organisms is scarce. In particular, the function of the *D. melanogaster* ortholog of CNDP2, encoded by the *CG17337* gene (hereafter referred to as *dCNDP2*), is still unknown.

**Results:**

This study was aimed at developing a set of genetic and molecular tools to study the roles of *dCNDP2*. We generated a *dCNDP2* null mutation (hereafter *∆dCNDP2*) using CRISPR/Cas9-mediated homologous recombination (HR) and found that the *∆dCNDP2* mutants are homozygous viable, morphologically normal and fertile. We also generated transgenic fly lines expressing eGFP-tagged and non-tagged dCNDP2 protein, all under the control of the UAS promoter, as well as polyclonal antibodies specific to dCNDP2. Using these tools, we demonstrate that only one of the two predicted dCNDP2 isoforms is expressed throughout the different tissues tested. dCNDP2 was detected in both the cytoplasm and the nucleus, and was found to be associated with multiple sites in the salivary gland polytene chromosomes.

**Conclusions:**

The *dCNDP2* gene is not essential for fly viability under standard laboratory conditions. The subcellular localization pattern of dCNDP2 suggests that this protein might have roles in both the cytoplasm and the nucleus. The genetic and molecular tools developed in this study will allow further functional characterization of the conserved CNDP2 protein using *D. melanogaster* as a model system.

**Electronic supplementary material:**

The online version of this article (10.1186/s12863-019-0726-z) contains supplementary material, which is available to authorized users.

## Background

Peptidases with different substrate specificities play distinct roles in protein and peptide metabolism in eukaryotes. Mammalian CNDP2 (also known as carnosine dipeptidase II, CN2, carboxypeptidase of glutamate-like, CPGL) belongs to the M20 family of metallopeptidases and has broad substrate specificity for dipeptides [1, 2]. It is active only as a homodimer [3], and the catalytic domain of each dimer subunit has one active center with two Mn^2+^ or Zn^2+^ ions, which determine the specificity of the enzyme for its physiological substrates [4]. So far, CNDP2 is the only known protease that can catalyze the formation of pseudodipeptides of lactic acid and amino acids (*N*-lactoyl-amino acids) through reverse proteolysis in vivo [5]. Thus, besides its proteolytic activity, CNDP2 might perform other cellular functions.

An aberrant expression of *CNDP2* is associated with tumorigenesis in humans. A decreased CNDP2 level was observed in pancreatic cancer, hepatocellular carcinoma and gastric cancer [2, 6, 7]. The CNDP2 isoform, CPGL-B, which lacks part of the catalytic domain, has been also implicated in tumor suppression, but it is currently unknown whether CPGL-B has peptidase activity [2, 6, 7]. However, not all tumors are characterized by low CNDP2 levels. An upregulated expression of CNDP2 has been indeed observed in breast carcinoma, and in kidney and colon cancers [8–11]. Several studies have been devoted to understanding the contribution of *CNDP2* to carcinogenesis [2, 6–11], but an animal model with mutated *CNDP2* is not currently available.

The CNDP2 protein is highly conserved across species [1, 12–14] and ubiquitously expressed ([1]; A Database of *Drosophila* Genes & Genomes available from ﻿ flybase.org [15]). In mouse and human cells, CNDP2 localizes to the cytosol and the nucleoplasm (Human Protein Atlas available from www.proteinatlas.org [16]); in transiently transfected Chinese hamster ovary (CHO) cells, CNDP2 was found in the cytoplasmic fraction [1]. In *D. melanogaster*, there is only one *CNDP2* gene (*CG17337;* flybase.org [15]), hereafter referred to as *dCNDP2*. Alignment of the amino acid sequences of the longest isoform of human CNDP2 (475 amino acids; GenPept accession no. NP_060705.2) and the longest isoform of *Drosophila* dCNDP2 (478 amino acids; GenPept accession no. NP_610181.2) revealed 63% sequence identity along the entire length of the polypeptide. Among the few *D. melanogaster* genes encoding the M20 metallopeptidase proteins, only *dCNDP2* is ubiquitously expressed with moderate to high level (flybase.org [15]). The product of this gene was shown to be an extracellular component of larval hemolymph [17, 18], but its subcellular localization is unknown.

To address the role of CNDP2 in cell growth and proliferation we decided to exploit *D. melanogaster* as a model system. The ease of genomic manipulations in flies allows investigation of processes at both the organismal and cellular levels. In this study, we generated a null *dCNDP2* mutant, transgenic lines for inducible *dCNDP2* expression, polyclonal antibodies against dCNDP2 and use these tools to provide an initial characterization of the protein.

## Methods

### Fly stocks

Flies were raised and crossed at 25 °C according to standard procedures. The following lines from the Bloomington Drosophila Stock Center (Bloomington, IN; bdsc.indiana.edu) were used: #1824 (*y*^*1*^ w*; P{w^*+mW.hs*^*=GawB}AB1*); #32186 (w*; *P{y*^*+t7.7*^*w*^*+mC*^*=10xUAS-IVS-mCD8::GFP}attP40*); #4775 (*w*^*1118*^; *P{w*^*+mC*^*=UAS-GFP.nls}14*); #24488 (*y*^*1*^ M{vas-int.Dm}ZH-2A w*; *M{3xP3-RFP.attP}ZH-102D*); #51325 (*w*^*1118*^; *PBac{y*^*+mDint2*^*=vas-Cas9,U6-tracrRNA}VK00027*) and #6599 (*y*^*1*^ *w*^*67c23*^) as a wild-type control. The stock carrying the *nanos-Cre* transgene [19] and the *P{CaryP}attP154* line [20] were kindly provided by Stepan N. Belyakin and Sergei A. Demakov, respectively (IMCB SB RAS, Novosibirsk, Russia).

### Plasmid constructs

Twenty-nucleotide guide RNA (gRNA) sequences for the *dCNDP2* gene, gRNA1 (5′-GUAAAAUAGAUUCGACGUAA-3′) and gRNA2 (5′-GAACCAGAUAUGACCCGCGA-3′), were designed using the CRISPR Design tool (zlab.bio/guide-design-resources). Their corresponding DNA sequences were cloned into the pU6-BbsI-chiRNA plasmid vector [21] downstream of the *Drosophila* U6 promoter by using the BbsI restriction sites to produce pU6-5′dCNDP2-chiRNA and pU6-3′dCNDP2-chiRNA constructs. Each plasmid expresses chimeric RNA (chiRNA) composed of the tracrRNA from *Streptococcus pyogenes* with the specific 20-nt gRNA sequence at the 5′ end [21]. The plasmid vector pU6-BbsI-chiRNA was a gift from Melissa Harrison & Kate O’Connor-Giles & Jill Wildonger (Addgene plasmid #45946).

The plasmid construct pGX-5′&3′-dCNDP2-null containing a “GMR enhancer-mini-*white* gene” reporter cassette placed between two DNA fragments, which flank the *dCNDP2* gene in the *D. melanogaster* genome and here referred to as 5′- and 3′-homology arms (or simply the left and right arms), was made as follows. First, the left arm (2R:5,695,002–5,696,806; here and afterwards, coordinates are from Release 6 of the *D. melanogaster* genome assembly [22]) was cloned into the pGX-attP plasmid vector [23] using the unique NotI and KpnI sites. This yielded the intermediate pGX-5′-dCNDP2-null plasmid. Next, the right arm (2R:5,701,122–5,703,566) was cloned into the pGX-5′-dCNDP2-null plasmid by using the unique AscI and XhoI sites to produce the pGX-5′&3′-dCNDP2-null construct. The cloned right arm contains the following three single nucleotide variations, all upstream of the *vlc* gene transcription start site: 5,701,650 T > C, 5,701,704_5,701,705insA and 5,701,862 T > C. The plasmid vector pGX-attP [23] was kindly provided by Sergei A. Demakov (IMCB SB RAS, Novosibirsk, Russia).

To make a rescue construct pGE-attB-GMR-dCNDP2, we cloned a 4.3-kb genomic DNA fragment carrying the *dCNDP2* gene (2R:5,696,807–5,701,121) into the pGE-attB-GMR plasmid vector [23] using the unique NheI and AscI sites. The cloned DNA fragment contains the following eight single nucleotide variations within and nearby the *dCNDP2* gene: 5,697,584C > T (upstream of the distal transcription start site), 5,698,421 T > A, 5,699,251 T > G, 5,699,713G > T, 5,699,725del (in the intronic regions), 5,699,463G > A (synonymous substitution), 5,700,144 T > A (in the 3′ untranslated region) and 5,700,278 T > C (downstream of the transcription termination site). The plasmid vector pGE-attB-GMR [23] was kindly provided by Sergei A. Demakov (IMCB SB RAS, Novosibirsk, Russia).

To generate a pUASTattB-dCNDP2 construct for ectopic expression of the dCNDP2 protein, we first PCR-amplified the DNA sequence (corresponding to nucleotides 100–1536 of GenBank accession no. NM_136337.3, but with 1023G > A and 1154A > T nucleotide substitutions) encoding the longer isoform of dCNDP2 (478 amino acids; hereafter dCNDP2-A). As a template, we used a cDNA synthesized from total RNA isolated from 0 to 24 h embryos from the wild-type Canton-S strain. Next, the amplified DNA fragment was cloned into the pUASTattB plasmid vector [24] by using the unique EcoRI and XbaI sites.

To make pUASTattB-eGFP-dCNDP2 and pUASTattB-dCNDP2-eGFP constructs for ectopic expression of N- and C-terminal eGFP-tagged dCNDP2 fusion proteins, we used the full-length *dCNDP2-A* coding sequence (see above). It was fused in-frame either downstream or upstream of the *eGFP* coding sequence using site-directed mutagenesis by overlap extension [25]. The DNA fragments were then cloned into the pUASTattB plasmid vector [24] using the unique EcoRI and XbaI sites and the EcoRI and KpnI sites, respectively.

To produce the pGEX-4 T-dCNDP2 construct, the full-length *dCNDP2-A* coding sequence (see above) was cloned in-frame into the pGEX-4 T-1 plasmid vector (GE Healthcare) downstream of the *glutathione S-transferase* (*GST*) coding sequence using the BamHI and XhoI sites.

All plasmid constructs were verified by DNA sequencing. Details of plasmid constructions are available upon request.

### Germline genome editing and transgenesis

To generate a null allele of *dCNDP2*, the pU6-5′dCNDP2-chiRNA and pU6-3′dCNDP2-chiRNA targeting constructs and the donor pGX-5′&3′-dCNDP2-null construct, all dissolved in water, were mixed to final concentrations of 125 ng/μl, 125 ng/μl and 500 ng/μl, respectively. The mixture was injected into embryos of the *w*^*1118*^; *PBac{y*^*+mDint2*^ *= vas-Cas9,U6-tracrRNA}VK00027* strain (Bloomington stock #51325) according to the standard procedure as previously described [26]. In total, 986 embryos were injected, out of which only 191 (19.4%) developed to the adult stage. Transformants were identified in the G_1_ progeny of fertile injectees by *w*^*+*^ phenotype. We found only one such fly male, indicating that the efficiency of the CRISPR/Cas9-mediated HR at the *dCNDP2* locus was 0.1% (1/986). The pGE-attB-GMR-dCNDP2 plasmid was injected at the concentration of 300 ng/μl into *∆dCNDP2* embryos expressing the phiC31 integrase in the germline [24]. The pUASTattB-dCNDP2, pUASTattB-eGFP-dCNDP2 and pUASTattB-dCNDP2-eGFP plasmids were individually injected at the concentration of 300 ng/μl into embryos carrying attP154 landing site [20] and expressing the phiC31 integrase in the germline [24].

### Genomic DNA extraction and PCR genotyping of engineered *dCNDP2* alleles

To isolate genomic DNA, 3–5 flies were grinded in 200 μl of DNA extraction buffer (100 mM Tris-HCl [pH 7.5], 100 mM NaCl, 0.5% SDS, 50 mM EDTA [pH 8.0], 200 mM sucrose) in an 1.5-ml tube and the sample was incubated at 65 °C for 30 min. Next, 300 μl of 5 M KAc were added, mixed well by inversion and the sample was kept on ice for 30 min. The tube was centrifuged for 15 min at 14,000 rpm. Then, the supernatant was transferred into a new tube and the DNA was precipitated with ethanol. Finally, the pellet was dissolved in 10–50 μl of nuclease-free water. PCR was performed using Hot-Start *Taq* DNA polymerase (Biolabmix, MH010) according to the manufacturer’s recommendations. For genotyping the *dCNDP2* locus after each step of its modification, allele-specific primer pairs were used (Additional file 1: Table S1). The PCR products were analyzed on 1% agarose gel along with GeneRuler 1 Kb Plus DNA Ladder (Thermo Scientific, SM1331).

### Anti-dCNDP2 antibody production

The GST-dCNDP2 fusion protein was expressed in *Escherichia coli* strain BL21(DE3)pLysS (Promega) and subsequently purified as described previously [27]. The purified GST-dCNDP2 fusion protein was used to immunize mice. Polyclonal antibodies were affinity purified from serum as reported earlier [27].

### S2 cell culture and RNA interference (RNAi)

First, we selected an 826-bp *dCNDP2* gene fragment, which is present in both transcript isoforms of the gene, as a template for the synthesis of double-stranded RNA (dsRNA). We amplified the DNA fragment using primers 5′-TAATACGACTCACTATAGGGAGGcgagatcggtcg-3′ and 5′-TAATACGACTCACTATAGGGAGGatagcgccacctgg-3′ that contain the T7 polymerase promoter sequence at their 5′ ends (shown in capital letters). The PCR product was purified using the GeneJET PCR Purification Kit (Thermo Scientific, K0702) and then used as a template to synthesize dsRNA as described earlier [28], with minor modifications. Treatment with DNaseI was done after heating the synthesized dsRNA to 65 °C and its slow cooling to room temperature; in addition, the phenol/chloroform extraction was omitted.

S2 cells were free from mycoplasma contamination and were cultured in 39.4 g/L Shields and Sang M3 Insect medium (Sigma, S8398) supplemented with 0.5 g/L KHCO_3_ and 20% heat-inactivated fetal bovine serum (FBS; Thermo Scientific, 10270106) at 25 °C. RNAi treatments were carried out as described previously [29], with the following modifications. Twenty-five μg of purified dsRNA was added to the cells three times (on the first, the third and the fifth days of incubation) and cells were harvested for analyses after 7 days of RNAi. Control S2 cell samples were prepared in the same way, but without addition of dsRNA.

### Western blotting

Subcellular fractionation of normal S2 cells was conducted as described previously [30]. RNAi-treated and control S2 cells were harvested by centrifugation at 200 *g* for 5 min at room temperature, washed with phosphate-buffered saline (PBS) and centrifuged again. S2 cell pellets and *Drosophila* embryos were homogenized and lysed in RIPA buffer (Sigma, R0278) containing 1× Halt™ Protease and Phosphatase Inhibitor Cocktail (Thermo Scientific, 1861282). The lysates were clarified by centrifugation at 15,000 *g* for 15 min at 4 °C and the protein extracts were normalized using the DC Protein Assay (Bio-Rad, 5000116). Each normalized sample was mixed with an equal volume of 2× Laemmli buffer and incubated for 5 min at 95 °C prior to analysis by SDS-PAGE and subsequent immunoblotting. Dissected larval and fly tissues were homogenized in 1× Laemmli buffer and incubated at 95 °C for 5 min prior to analysis by SDS-PAGE and subsequent immunoblotting. The primary antibodies were mouse α-Lamin Dm0 (1:300; Developmental Studies Hybridoma Bank, ADL67.10), mouse α-alpha-tubulin (1:5000; Sigma, T6199), mouse α-dCNDP2 antibody (1:10,000; this study), rabbit α-Histone H3 (1:1000; Pierce, PA5-17697) and rabbit α-GFP (1:2000; Sigma, G1544); they were detected using HRP-conjugated goat α-mouse IgG (1:3500; Life Technology, G-21040) and goat α-rabbit IgG (1:3500; Life Technology, G-21234). Images were captured using an Amersham Imager 600 System (GE Healthcare).

### Immunofluorescence (IF) staining

Immunostaining of S2 cells and squashed salivary gland polytene chromosomes was performed as described previously [31, 32]. The primary mouse polyclonal α-dCNDP2 antibodies were used at dilution 1:1000 and detected by goat α-mouse IgG antibodies conjugated to Alexa Fluor 488 (1:500; Invitrogen, A-11001). IF images of S2 cells were made using Zeiss LSM 710 confocal microscope and an oil 100×/1.40 plan apo lens. IF images of polytene chromosomes were acquired with a Zeiss Axio Observer.Z1 fluorescence microscope equipped with an Axiocam 506 mono (D) camera using an oil 63×/1.40 plan apo lens. In both cases, ZEN 2012 software was used for image acquisition.

For detection of eGFP-tagged dCNDP2 fusion proteins, whole salivary glands were fixed in PBS containing 4% formaldehyde (Merck, 104003), washed 3 times for 5 min each with PBS containing 0.5% Triton X-100, stained for 30 min with 0.4 μg/ml DAPI dissolved in PBS and mounted in 50% Glycerol dissolved in PBS. IF images of whole-mounted glands were obtained on a Zeiss LSM 710 confocal microscope using a 20×/0.50 EC Plan-Neofluar lens. Optical sections were combined using the LSM Image Browser version 3.5 software (Zeiss).

## Results

**Fig. 1:**
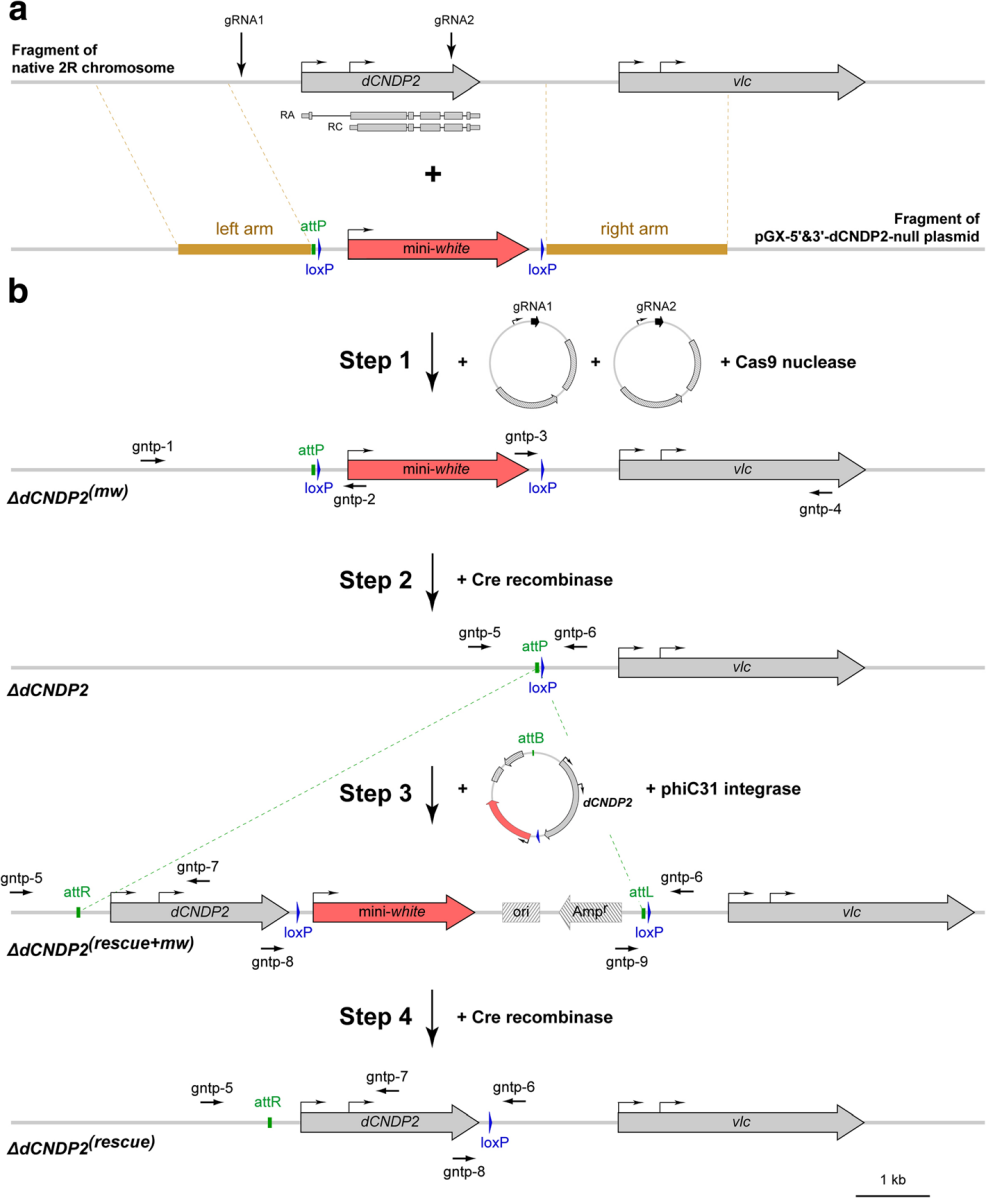
Experimental design for generation of null and rescue alleles of the *dCNDP2* gene. **a** Schematic representation of the *dCNDP2* locus and the donor plasmid pGX-5′&3′-dCNDP2-null, which contains the mini-*white* reporter gene (red) flanked by the left and right homology arms (brown). The gRNA target sites are indicated by vertical arrows. **b** Modifications introduced into the *dCNDP2* locus. In step 1, the *dCNDP2* gene was replaced by the mini-*white* reporter gene using CRISPR/Cas9-mediated HR approach. Note that the reporter gene is flanked by two loxP sites. In step 2, the mini-*white* reporter was removed by Cre recombinase-mediated recombination between loxP sites. This resulted in generation of a null allele (∆*dCNDP2*), in which the *dCNDP2* gene is replaced by the attP and loxP sites. In step 3, the rescue construct pGE-attB-GMR-dCNDP2 was integrated into the attP site by phiC31 integrase-mediated recombination. The rescue construct contains the mini-*white* reporter gene, which upon integration into the genome becomes flanked by two loxP sites, and the genomic DNA fragment carrying the *dCNDP2* gene that is absent in ∆*dCNDP2* mutants. In step 4, the mini-*white* reporter was removed by Cre recombinase-mediated recombination between loxP sites. This resulted in generation of the *∆dCNDP2*^*(rescue)*^ transgenic flies, in which DNA sequence of the *dCNDP2* locus is almost identical to the wild-type one except for several single nucleotide variations (see Methods) and the presence of the attR and loxP sites. Black horizontal arrows indicate primers used for PCR genotyping (for primers sequences, see Additional file 1: Table S1). Plasmids are drawn as circles with the relevant elements indicated; Amp^r^, ampicillin resistance gene; ori, plasmid origin of replication

To address the function of *dCNDP2*, we generated a null mutation in the gene, *∆dCNDP2*, using the recently developed CRISPR/Cas9-mediated HR method [21], which is illustrated in detail in Fig. 1 and in Additional file 2: Figure S1. The final product of this procedure was the *∆dCNDP2* null mutation that carries a phiC31 attP site instead of the ~ 4.3-kb DNA sequence containing the entire *dCNDP2* gene. We found that homozygous mutants do not display visible morphological abnormalities and are fertile. We also used phiC31 integrase-mediated recombination to deliver the same ~ 4.3-kb DNA fragment that was deleted in *∆dCNDP2* back into its original genomic location, and generated the *∆dCNDP2*^*(rescue)*^ allele (Fig. 1; Additional file 2: Figure S1).

**Fig. 2:**
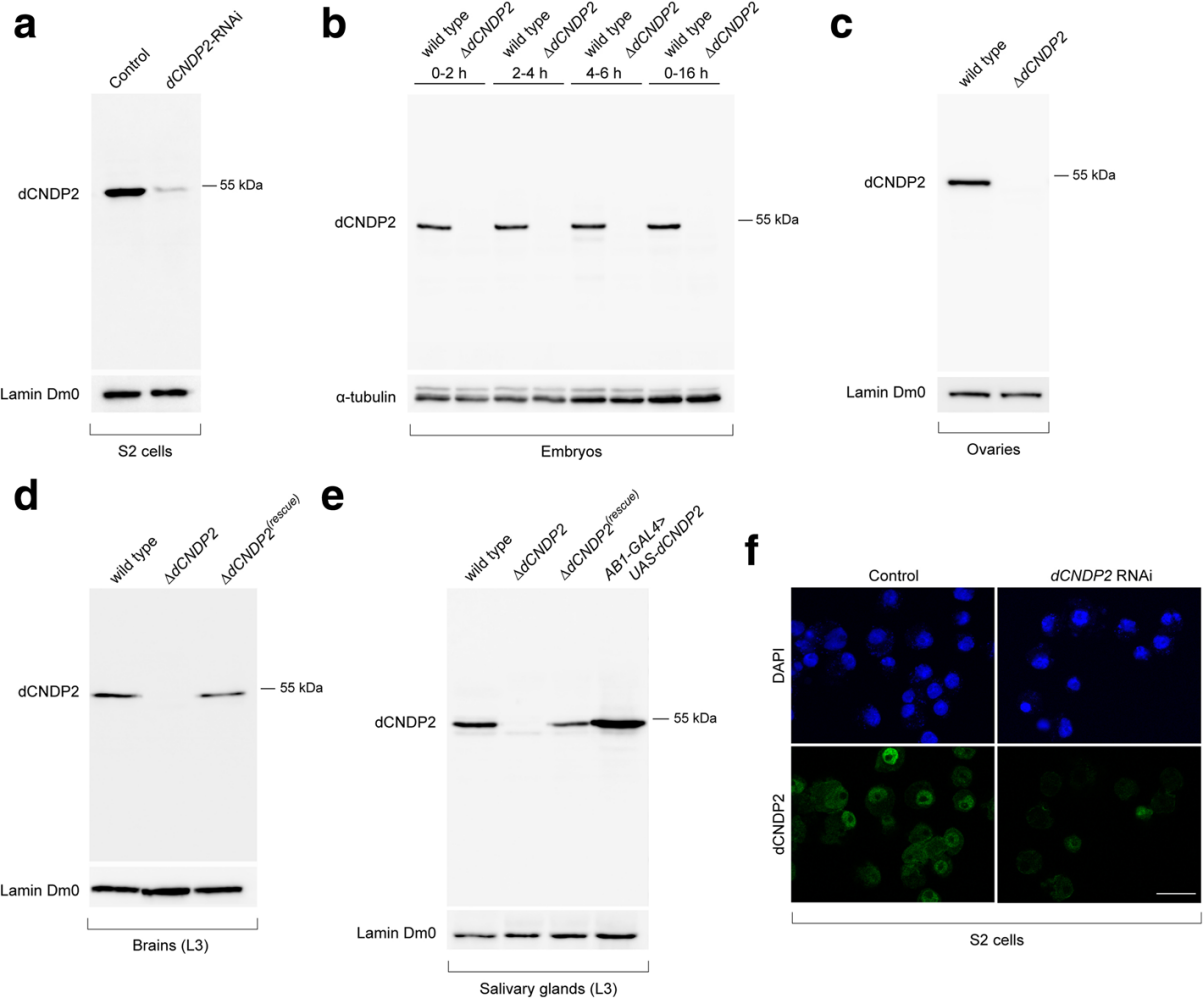
α**-**dCNDP2 antibodies are specific to the target protein. **a**-**e** Western blots showing that the α**-**dCNDP2 antibodies recognize a single band migrating slightly lower than the 55 kDa standard. This band is likely to correspond to the 53.2 kDa isoform A. **a** S2 cells; **b** embryos; **c** ovaries; **d** larval brains; **e** salivary glands. L3, third instar larval stage. Note that in 4–6 h old embryos the antibodies recognize a second faint band that might correspond to the 47.9 kDa isoform C. **f** Immunostaining of dCNDP2 depleted (*dCNDP2* RNAi) and control S2 cells with α**-**dCNDP2 antibodies. Scale bar, 20 μm

The wild-type *dCNDP2* gene is predicted to encode two protein isoforms that are slightly different in their amino acid sequences (the A and C isoforms, with molecular weights of 53.2 kDa and 47.9 kDa, respectively; see flyBase [15]). Namely, the shorter isoform does not contain the first 47 amino acids that are present at the N-terminus of the longer isoform. To analyze the expression pattern of dCNDP2 protein isoforms during *Drosophila* development, we generated antibodies using the full-length dCNDP2-A isoform as an antigen. The specificity of the antibodies was first assessed by Western blotting of protein extracts prepared from normal S2 cells and S2 cells treated with *dCNDP2* dsRNA. In control cells, we observed a clear signal of the expected molecular weight that was almost absent in RNAi cells (Fig. 2a). Western blotting with α-dCNDP2 antibodies showed the absence of the protein also in embryos, ovaries, brains and salivary glands of *∆dCNDP2* mutants (Fig. 2b–e), and the expected restoration of dCNDP2 expression level in *∆dCNDP2*^*(rescue)*^ flies (Fig. 2d, e). Interestingly, in most tissues examined, the antibodies recognized a single band, presumably corresponding to the dCNDP2-A isoform (Fig. 2b–e). Only in 4–6 h old embryos we detected a second faint band that might correspond to the smaller predicted isoform of dCNDP2 (Fig. 2b). These results suggest that only one of the two predicted isoforms of the dCNDP2 protein is expressed in most tissues.

**Fig. 3:**
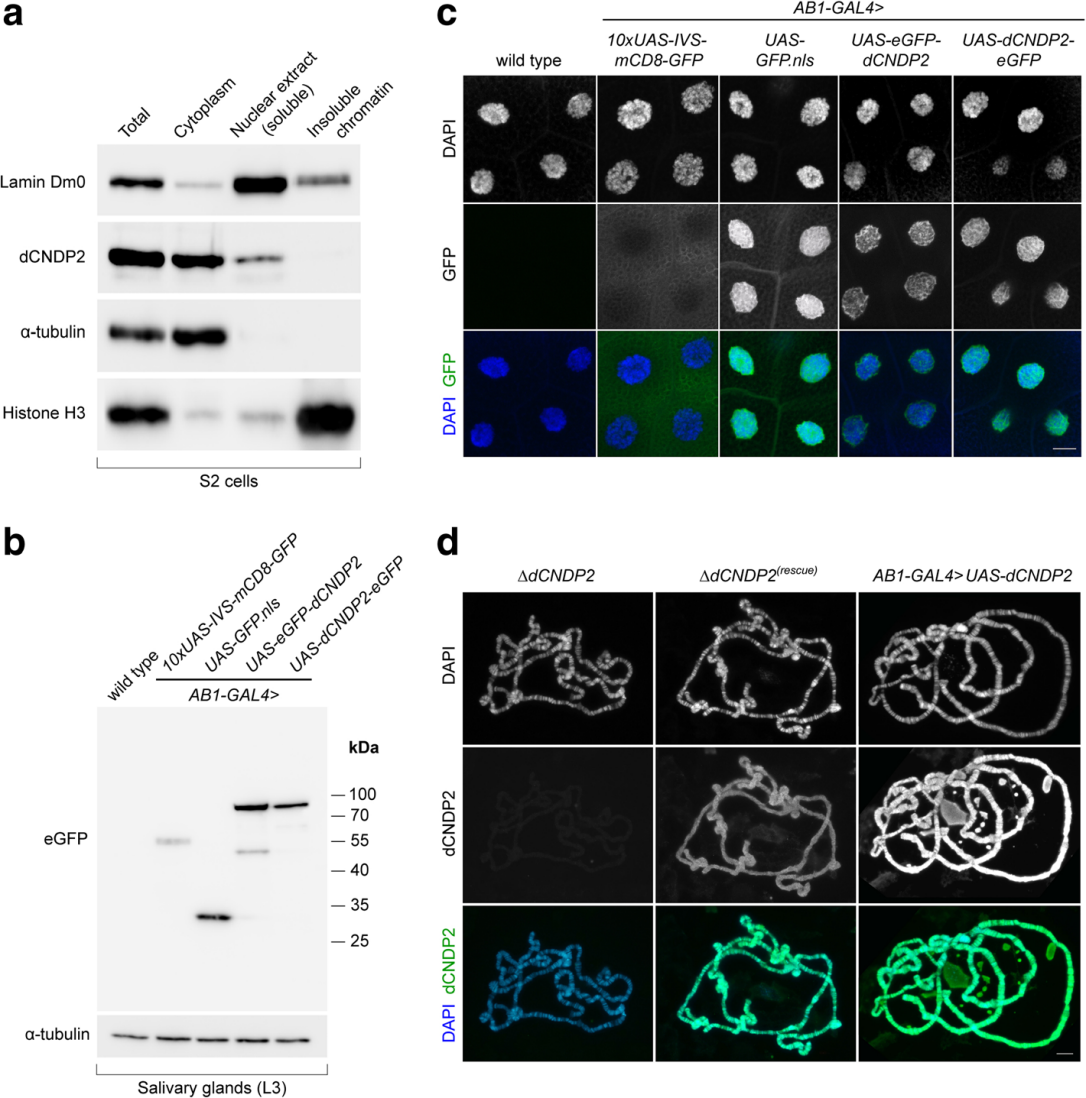
dCNDP2 is present in both the cytoplasm and the nucleus. **a** Western blot showing subcellular fractionation of S2 cells. **b** Western blot showing ectopic expression of eGFP-tagged proteins in salivary glands using the *AB1-GAL4* driver. L3, third instar larval stage. **c** Localization of eGFP-tagged proteins in L3 salivary gland cells. GFP.nls and mCD8:GFP were used as controls for nuclear localization, and cytoplasmic and transmembrane localization, respectively. Scale bar, 20 μm. **d** dCNDP2 binds multiple sites on polytene chromosomes. The following exposure times were used to acquire images of chromosomes immunostained with α**-**dCNDP2 antibodies: 800 msec, *∆dCNDP2* and *∆dCNDP2*^*(rescue)*^; 500 msec, *AB1-GAL4 > UAS-dCNDP2*. Scale bar, 20 μm

To determine the subcellular localization of dCNDP2, we first immunostained S2 cells with α-dCNDP2 antibodies. We found that the protein is present in both the nucleus and the cytoplasm, and that the nuclei exhibit an unstained region that could be the nucleolus (Fig. 2f). Consistent with Western blotting analysis, both the nuclear and cytoplasmic stainings were dramatically reduced after the depletion of the protein by RNAi (Fig. 2f). The localization pattern of dCNDP2 was confirmed by subcellular fractionation of S2 cell extracts. This analysis revealed that the protein is predominantly cytoplasmic, but it is also a soluble nuclear component (Fig. 3a).

We also generated transgenic fly lines expressing N- or C-terminally eGFP-tagged dCNDP2 under the control of the UAS promoter. When eGFP-dCNDP2 and dCNDP2-eGFP proteins were expressed using *AB1-GAL4* driver, both recombinant proteins were predominantly localized within the nucleus of salivary gland cells of third instar larvae (Fig. 3b, c). We then immunostained salivary gland polytene chromosomes from *∆dCNDP2*, *∆dCNDP2*^*(rescue)*^ and *AB1-GAL4 > UAS-dCNDP2* larvae with α-dCNDP2 antibodies and found that dCNDP2 associates with multiple chromosomal sites (Fig. 3d). These results indicate that the *Drosophila* dCNDP2 protein, as its mammalian counterpart, is a component of both the cytoplasm and the nucleus. In addition, dCNDP2 protein is able to bind the chromatin of polytene chromosomes.

## Discussion

Our knowledge on consequences of the complete loss of the *CNDP2* gene function in humans is very limited. There is only a single case report of a likely complete deficiency of this gene, which is associated with global developmental delays, ataxia, hypotonia and tremor [33]. Yet, in this case, the presence of a gross chromosomal deletion does not allow a firm conclusion on whether the effects are caused solely by *CNDP2* deficiency [33]. Thus, it is difficult to decide whether the phenotypic consequences of *Drosophila dCNDP2* loss are substantially different from those elicited by the loss of the human homolog.

Many studies reported that the *CNDP2* gene is misexpressed in different types of human cancers [2, 6–11]. CNDP2 was also found to be involved in the regulation of the cell cycle in human cancer cell lines. Specifically, CNDP2 overexpression in pancreatic cancer lines induced the accumulation of cells in the G0/G1 phase [6], whereas knockdown of CNDP2 in colon cancer cells blocked the cell cycle progression in the G2/M phase [11]. However, the molecular mechanisms underlying these effects have not been uncovered. Thus, it will be interesting investigating whether dCNDP2 is implicated in the control of the *Drosophila* cell cycle.

The binding of dCNDP2 to polytene chromosomes suggests that it may be involved in the regulation of processes such as DNA replication, transcription or repair. However, we have limited information on how this protein is transported into the nucleus. Typically, the nuclear localization of a protein larger than 40–45 kDa is controlled by the presence of a nuclear localization signal (NLS) of a specific amino acid sequence (for review, see [34–36]). Indeed, we predicted an importin-α/β pathway-specific monopartite NLS (YWLGKKRPCLTY) in the middle portion of the dCNDP2 protein, using the cNLS Mapper (nls-mapper.iab.keio.ac.jp/cgi-bin/NLS_Mapper_form.cgi [37]). However, the functionality of this motif as a NLS has not been tested so far. A definition of the dCNDP2 NLS could help designing experiments aimed at understanding of the role of this protein in the nucleus.

## Conclusions

We developed a set of genetic and molecular tools to study the function of the dCNDP2 protein in *D. melanogaster*. Particularly, we generated the *dCNDP2* null allele using a CRISPR/Cas9-mediated HR approach and different transgenic flies to overexpress *dCNDP2*. We demonstrated that this gene is not essential for fly viability under standard laboratory conditions. Although two dCNDP2 isoforms with different molecular weights have been previously predicted, we detected the presence of only one of them in most *Drosophila* tissues. Moreover, we found that dCNDP2 is localized not only in the cytoplasm, but also in the nucleus, where it can be associated with chromatin. Thus, both the amino acid sequence of the protein and its subcellular localization are conserved between mammals and fruit flies. This indicates that *D. melanogaster* is a good model for further elucidation of the mechanisms of CNDP2 action in the context of the whole organism.

## Additional files

Additional file 1: **Table S1.** Primers used for PCR genotyping of *dCNDP2* alleles and PCR product details. (PDF 139 kb)

Additional file 2: **Figure S1.** Molecular verification of *dCNDP2* alleles. (PDF 553 kb)

## Abbreviations

*∆dCNDP2*: *dCNDP2* null mutation
Cas9: CRISPR associated protein 9
chiRNA: Chimeric RNA
CHO cells: Chinese hamster ovary cells
CN2: Carnosine dipeptidase II
CNDP2: Cytosolic non-specific dipeptidase 2
CPGL: Carboxypeptidase of glutamate-like
CRISPR: Clustered regularly interspaced short palindromic repeats
DAPI: 4′,6-diamidino-2-phenylindole
dCNDP2: Drosophila CNDP2
dsRNA: Double-stranded RNA
eGFP: Enhanced green fluorescent protein
FBS: Fetal bovine serum
GMR: Glass multiple reporter
gRNA: Guide RNA
HR: Homologous recombination
HRP: Horseradish peroxidase
IF: Immunofluorescence
NLS: Nuclear localization signal
PBS: Phosphate-buffered saline
RNAi: RNA interference
SDS-PAGE: Sodium dodecyl sulphate-polyacrylamide gel electrophoresis
tracrRNA: Trans-activating crRNA
UAS: Upstream activation sequence
