# Supplementary material for: A toolset to study functions of Cytosolic non-specific dipeptidase 2 (CNDP2) using Drosophila as a model organism
Source: BMC Genet. 2019 Mar 18;20(Suppl 1):31. doi: 10.1186/s12863-019-0726-z (PMC6421639; doi:10.1186/s12863-019-0726-z)
Supplement: Supplementary file 1 — Table S1. Primers used for PCR genotyping of dCNDP2 alleles and PCR product details. (PDF 139 kb) [file 12863_2019_726_MOESM1_ESM.pdf]

Table S1. Primers used for PCR genotyping of *dCNDP2* alleles and PCR product details.

Primer name	Primer sequence (5′->3′)	Amplicon
gntp-1	ggagattgccttctcaataaagtgg	no product from wild-type flies
gntp-2	gtacagagagggagagtcac	2672 bp from homozygous *ΔCNDP2*$^{(mw)}$ mutant flies
gntp-3	aaactcgacaccggtataacttc	no product from wild-type flies
gntp-4	cgttgtactctgagaacacttcttctg	2800 bp from homozygous *ΔCNDP2*$^{(mw)}$ mutant flies
gntp-5	ttttggcaggtaaacaataaaacg	5457 bp from wild-type flies<br>4332 bp from homozygous *ΔCNDP2*$^{(mw)}$ mutant flies
gntp-6	atttagctgttttccagacgatag	1271 bp from homozygous *ΔdCNDP2* mutant flies
gntp-5	ttttggcaggtaaacaataaaacg	2382 bp from wild-type flies<br>2467 bp from homozygous Δ*dCNDP2*$^{(rescue+mw)}$ flies
gntp-7	tccattccaccatacgaccg	2467 bp from homozygous Δ*dCNDP2*$^{(rescue)}$ flies
gntp-8	gacgaacaaatgaaaatctgctgtg	1354 bp from wild-type flies<br>7551 bp from homozygous Δ*dCNDP2*$^{(rescue+mw)}$ flies
gntp-6	atttagctgttttccagacgatag	1435 bp from homozygous Δ*dCNDP2*$^{(rescue)}$ flies
gntp-9	catgatgaaataacataaggtggtcc	no product from wild-type flies
gntp-6	atttagctgttttccagacgatag	992 bp from homozygous Δ*dCNDP2*$^{(rescue+mw)}$ flies
