# Supplementary material for: A toolset to study functions of Cytosolic non-specific dipeptidase 2 (CNDP2) using Drosophila as a model organism
Source: BMC Genet. 2019 Mar 18;20(Suppl 1):31. doi: 10.1186/s12863-019-0726-z (PMC6421639; doi:10.1186/s12863-019-0726-z)
Supplement: Supplementary file 2 — Figure S1. Molecular verification of dCNDP2 alleles. (PDF 553 kb) [file 12863_2019_726_MOESM2_ESM.pdf]

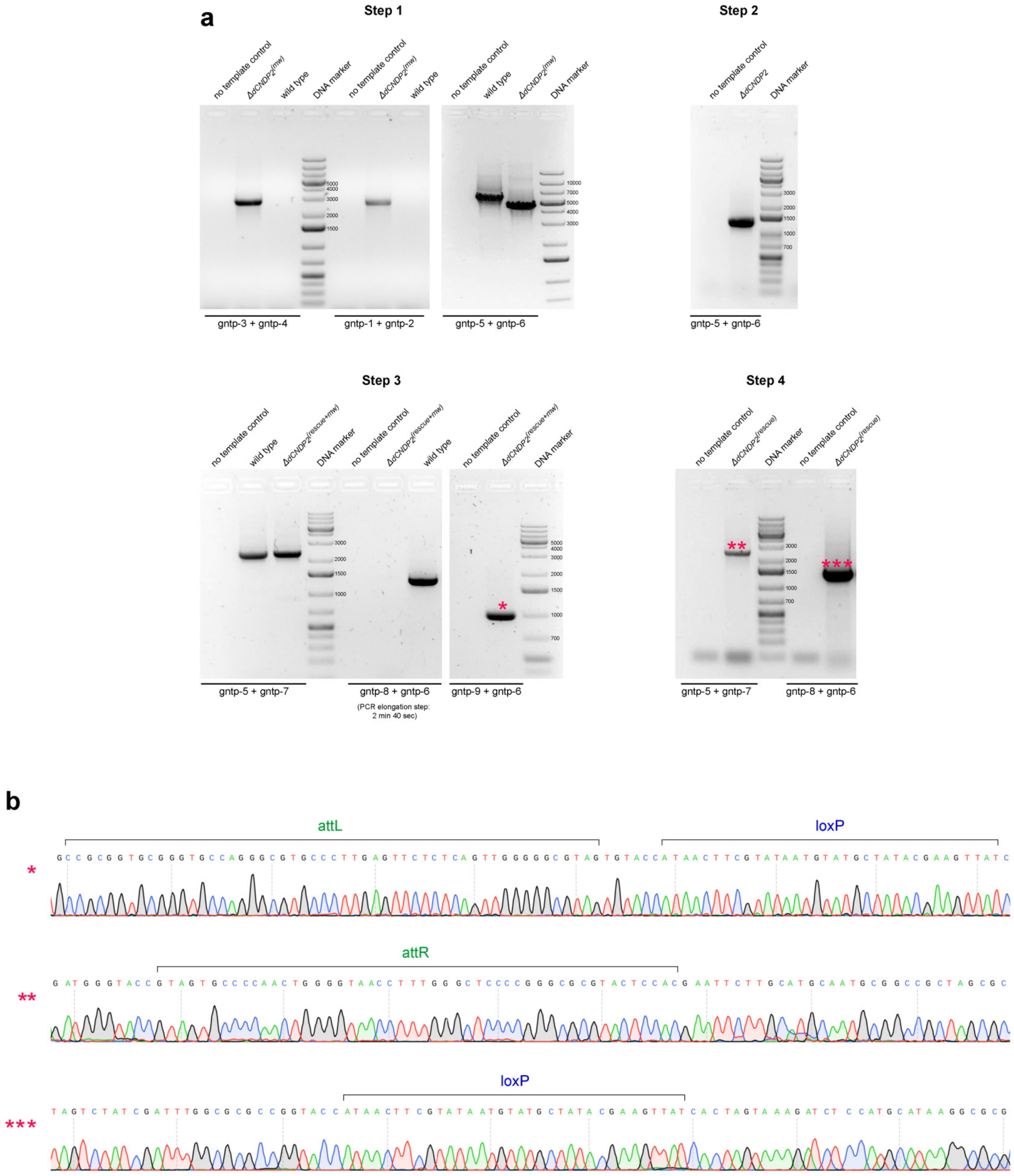

Figure S1. Molecular verification of *dCNDP2* alleles. **a** Agarose gel electrophoresis of PCR products obtained by using a set of allele-specific primer pairs (primers sequences are provided in Table S1) and DNA isolated from the indicated fly lines and water (as a negative control) as template. For the explanation of the steps 1-4, see Fig. 1b. Asterisks indicate PCR products that were partially sequenced. **b** Fragments of sequencing chromatograms of the indicated PCR products.
